# Untargeted Metabolomics Reveals Region-Specific Metabolic Signatures and Discriminative Markers in Goji Berry (*Lycium barbarum* L.)

**DOI:** 10.3390/metabo16050326

**Published:** 2026-05-14

**Authors:** Yan Yan, Wei Ma, Yage Li, Chen Zhang, Fang Li, Tianqing Huang, Beibei Gao, Huihui Meng, Yunfei Hu, Huan Wu

**Affiliations:** 1 College of Chinese Medicine, Bozhou University, Bozhou 236800, China; 2022070005@bzuu.edu.cn (Y.Y.); 2016070038@bzuu.edu.cn (W.M.); 2024070014@bzuu.edu.cn (Y.L.); 2022070007@bzuu.edu.cn (C.Z.); 2022070017@bzuu.edu.cn (F.L.); 2024070016@bzuu.edu.cn (T.H.); gaobeibei@bzuu.edu.cn (B.G.); menghuihui2014@bzuu.edu.cn (H.M.); 2 Key Laboratory of Xin’an Medicine, Ministry of Education, Anhui University of Chinese Medicine, Hefei 230038, China

**Keywords:** goji berry (*Lycium barbarum* L.), untargeted metabolomics, plant stress resistance, discriminative markers, environmental factors

## Abstract

**Background/Objectives**: Goji berry (*Lycium barbarum* L.), renowned as a typical medicinal and edible plant, is mainly cultivated across four agroclimatic zones in China, including semi-arid, arid, monsoon, and high-altitude regions. Ningxia has long been recognized as the *daodi* production area for goji berries. However, the metabolic diversity of goji berries from other core cultivation regions and how these differences are shaped by local environments remain poorly understood. **Methods**: In this study, untargeted metabolomics was employed to comprehensively investigate the metabolic difference in goji across seven production regions. By integrating multivariate analysis with KEGG pathway enrichment (*p* < 0.05), 49 discriminative markers enriched in 10 key pathways were putatively identified, and their roles in plant stress tolerance were elucidated. In addition, we conducted targeted quantification of key bioactive components and antioxidant capacity. **Results**: Significant regional differences were revealed. Redundancy analysis further identified rainfall, temperature, and UV radiation as the key climatic drivers of this variation. **Conclusions**: These findings provide insights into the metabolic adaptation of goji to local environments and serve as a basis for further functional studies.

## 1. Introduction

Goji berry (*Lycium barbarum* L.), a member of the Solanaceae family, has been valued for over two millennia in traditional medicine and is now globally recognized as a popular functional food and dietary supplement, as documented in authoritative references such as the Chinese Pharmacopoeia, the American Herbal Pharmacopoeia, and the European Pharmacopoeia [1]. Extensive studies have demonstrated that goji berry contains various bioactive constituents, such as polysaccharides, flavonoids, carotenoids, low molecular weight soluble sugars, and vitamins [2,3,4]. These components play important roles in both plant physiology and health-promoting activities, including antioxidant, anti-inflammatory, and immunomodulatory benefits [5,6]. With expanding global demand, goji cultivation has spread across diverse agroclimatic zones in China, encompassing the semi-arid (Ningxia, Inner Mongolia, and Gansu), arid (Xinjiang), monsoon (Hebei), and high-altitude regions (Qinghai and Xizang).

Although plants often adjust their metabolic profiles in response to varied environmental conditions [7], the traditional concept of *daodi* (authentic producing region) has long established Ningxia goji as the quality benchmark, noted for its unique semi-arid climate and alkaline soils [8]. Previous studies on geographical variation in goji have primarily focused on a restricted geographical scope [9,10], targeted analysis of a limited number of compounds, or non-specific spectroscopic methods [10,11,12,13,14,15]. While studies have provided valuable insights into the impact of specific growing regions on goji metabolome and are important for identifying certain markers, few studies have comprehensively investigated the metabolic difference in goji from other core producing regions, and how both bioactive components and metabolic profiles are shaped by local environments have not been sufficiently explored.

To fill this gap, we employed UPLC-QTOF-MS/MS-based untargeted metabolomics, a powerful tool to characterize plant chemical diversity and to discover biomarkers linked to environmental stress responses [16], extending the geographical scope of previous work to seven cultivation regions. Through pairwise comparisons with Ningxia goji and KEGG pathway enrichment analysis, discriminatory markers associated with significant pathways were putatively identified. Their putative roles in plant stress adaptation, functional food applications, and geographical authentication were further discussed. In addition, we performed quantitative analysis of key bioactive components, including total flavonoids (TFC), *Lycium barbarum* polysaccharides (LBPs), low molecular weight soluble sugars (fructose, sucrose, and glucose), and antioxidant capacity, providing complementary data on the nutritional and functional traits of goji berries, and exploring their relationship with core environmental factors via redundancy analysis (RDA). The results provide new insights into the region-specific cultivation, quality evaluation, and stress-resilient breeding of goji berries.

## 2. Materials and Methods

### 2.1. Geographical Locations and Climate Conditions

**Figure 1:**
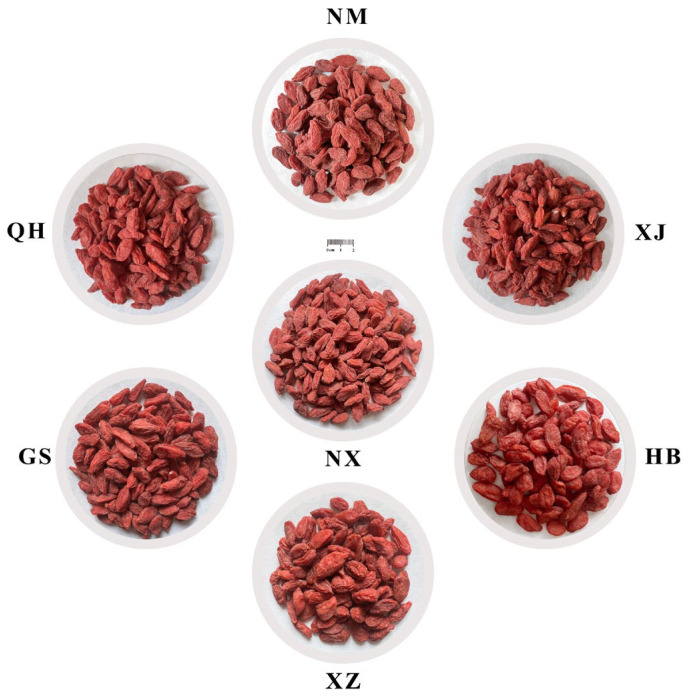
Representative morphological diversity of dried fruits per origin: NX (Zhongning, Ningxia), NM (Linhe; Bayannur; Inner Mongolia), GS (Yumen; Gansu), XJ (Jinghe; Xinjiang), QH (Golmud; Qinghai), XZ (Lhasa; Xizang), HB (Julu; Hebei).

Dried goji samples were collected in November 2023 from seven major cultivation areas in China. All samples were derived from conventionally managed, large-scale artificial cultivation. No organic or wild-harvested goji berries were included in this study. These samples had been processed by local cooperatives using traditional sun-drying methods immediately after harvest, during which the fruits were spread in thin layers and dried under natural sunlight for approximately 5–7 days, depending on weather conditions, until the moisture content was reduced to a commercially acceptable level (typically below 13%) to prevent mold growth and ensure storage stability. The sampling sites were selected as the representative core production zones for goji berries within each region, including Zhongning (Ningxia; NX), Linhe (Bayannur, Inner Mongolia; NM), Yumen (Gansu; GS), Jinghe (Xinjiang; XJ), Golmud (Qinghai; QH), Lhasa (Xizang; XZ), and Julu (Hebei; HB). Six independent batches were collected from each of the seven sampling sites. Their geographical coordinates were obtained via real-time positioning using Ovi interactive mapping software and are provided in Table 1. For visual comparison, 30 g of dried berries per origin were randomly sampled and arranged on a standardized background (Figure 1). All the samples were authenticated by Prof. MENG Xiangsong (Chief Traditional Chinese Medicine Pharmacist). Climate parameters during the active growth and fruit development phases (April–October 2023) were downloaded from the China Meteorological Data Service Center (http://data.cma.cn/, accessed on 15 May 2025). This period covered critical phenological stages for goji quality formation, as supported by previous studies [17]. Climatic variables included daily average, maximum and minimum temperatures, sunlight hours, rainfall, days with large diurnal temperature variations (≥17 °C), ultraviolet (UV) radiation, relative humidity (%), and accumulated temperature (≥10 °C), which are summarized in Table 1.

### 2.2. Bioactive Compounds and Antioxidant Capacity Analyses

LBP: According to the ChP (11th ed., 2025) [18], the content of LBP is determined using the phenol–sulfuric acid method. A calibration curve (0.01, 0.02, 0.03, 0.04, and 0.05 mg/mL) was prepared. Absorbance was measured at 490 nm using a spectrophotometer and the blank contained all reagents except glucose.

TFC: Approximately 0.5 g of ground goji was extracted with petroleum ether in a Soxhlet extractor until the extract became colorless. The ether layer was discarded and the residue was thoroughly dried in a fume hood. Ultrasonic extraction was then performed on the residue with 25 mL of 70% ethanol at 60 °C for 35 min. The ethanol extract was transferred to a 100 mL volumetric flask, diluted to volume with 70% ethanol and filtered. A calibration curve was made from rutin standard solution (0.01, 0.02, 0.04, 0.08, 0.15, and 0.20 mg/mL). Then, 6 mL of the standard or samples was taken and 1 mL of 5% NaNO_2_ was added and mixed for 6 min, and then 1 mL of 10% Al(NO_3_)_3_ was added and mixed for 6 min, followed by 10 mL of 4% NaOH. After dilution to 25 mL and incubation for 15 min, absorbance was measured at 500 nm against the blank reagent.

Low molecular weight soluble sugars (fructose, glucose, and sucrose): The extraction and quantification of soluble sugars (unified abbreviation of low molecular weight soluble sugars) were carried out as described by Lu et al. [19]. Briefly, goji powder (0.5 g) was ultrasonicated in 40 mL of deionized water (40 kHz, 30 °C, 60 min), centrifuged (15,000× *g*, 10 min), and filtered (0.45 μm). HPLC-ELSD analysis used a Prevail Carbohydrate ES column (4.6 × 250 mm, 5 μm) with a water–acetonitrile gradient (15–50% water in 20 min, 1.0 mL/min). ELSD conditions: 50 °C, 2.7 L/min N_2_. Calibration curves were prepared for fructose and glucose (0.5–5.0 mg/mL) and sucrose (0.05–0.8 mg/mL).

DPPH radical scavenging assay: This is a reliable and high-throughput assay that effectively quantifies antioxidant performance [20]. DPPH radical scavenging activity was measured using a total antioxidant capacity assay kit. In detail, 190 μL of DPPH solution was mixed with 10 μL of either the sample extract or vitamin C (l-ascorbic acid) standard solution (at concentrations of 0.000 [solvent blank], 0.031, 0.063, 0.125, 0.250, 0.300, and 1.000 mg/mL) in a 96-well microplate. The mixture was mixed well and incubated in the dark for 30 min. Then, the absorbance at 515 nm was determined and the scavenging activities were calculated as the percent inhibition according to the following formula: The scavenging rate of positive control (%) = [(A_1_ − A_2_) ÷ A_1_] × 100%; The scavenging rate of sample (%) = [[A_1_ − (A_3_ − A_4_)] ÷ A_1_] × 100%. The radical scavenging activity was calculated based on the following absorbance measurements: A_1_: blank (DPPH solution without test sample); A_2_: positive control; A_3_: test sample; A_4_: background control.

### 2.3. Untargeted Metabolomics Analysis

#### 2.3.1. Metabolites Extraction

Goji samples were freeze-dried under vacuum, pre-cooled by liquid nitrogen, and then homogenized into powder. After being sieved through an 80-mesh, approximately 0.2 g of goji powder was precisely weighed. Then, 1 mL of −20 °C pre-cooled extraction solvent methanol/acetonitrile/H_2_O (2:2:1, *v*/*v*/*v*) was added, yielding a sample-to-solvent ratio of 1:5 (*w*/*v*), and the mixture was vortexed for 1 min at 2500 rpm. After ultrasonic extraction at 4 °C for 30 min, the samples were kept at −20 °C for 10 min. Then, the extract was centrifuged at 14,000× *g* (4 °C) for 20 min, and the supernatant was collected and dried in vacuum. For UPLC-QTOF-MS analysis, the dried extracts were re-dissolved in 100 μL acetonitrile/water (1:1, *v*/*v*) and then centrifuged at 14,000× *g* (4 °C) for 15 min before analysis.

#### 2.3.2. UPLC-QTOF-MS Analysis

Chromatographic separation was performed by a UPLC (1290 Infinity LC, Agilent Technologies, Co., Ltd., Santa Clara, CA, USA) equipped with an ACQUITY UPLC BEH C18 column (1.7 μm, 2.1 mm × 100 mm, Waters, Milford, MA, USA). The mobile phase was composed of water containing 0.1% formic acid with 5 mM ammonium acetate (solvent A) and acetonitrile containing 0.1% formic acid (solvent B), with the gradient elution program set as follows: 0–0.5 min, 5% B; 0.5–10 min, 5–100% B; 10–12min, 100% B; 12–12.1 min, 100–5% B; 12.1–16 min, B was maintained at 5%. The column temperature was kept at 40 °C and the flow rate was set at 0.4 mL/min, with an injection volume of 2 μL. During the whole analysis, samples were kept at 4 °C and injected in a random sequence to minimize the impact of instrument fluctuations. To ensure system stability and reproducibility throughout the analytical run, a pooled quality control (QC) sample was prepared and strategically interspersed at intervals of approximately every ten samples within the sequence (*n* = 42 biological samples). System stability was evaluated using QC samples, with the acceptance criterion that more than 80 percent of the identified metabolic features must exhibit a relative standard deviation (RSD) of 30 or less [21].

The MS/MS analysis of goji was performed using a QTOF-MS system (AB Sciex TripleTOF 6600, AB Sciex Pte. Ltd., Framingham, MA, USA). The ESI source parameters were set as follows: Ion Source Gas1 and Ion Source Gas2 were set at 60 psi, curtain gas (CUR) at 30 psi, source temperature at 600 °C, and ion spray voltage ± 5500 V in both positive and negative ESI modes. The mass range of precursor ion scanning was *m*/*z* from 60 to 1200 Da and MS/MS product ion scanning from 25 to 1000 Da. The accumulation time for TOF MS scan was set at 0.20 s/spectra and product ion scan was 0.05 s/spectra. Information-dependent acquisition (IDA) with a high sensitivity mode was performed to scan the product ion and the parameters were as follows: the collision energy (CE) was 35 with ± 15 eV; declustering potential (DP) was set at 60 V (negative and positive mode); exclude isotopes within 4 Da; and 10 candidate ions were monitored per cycle.

### 2.4. Data Processing and Statistical Analysis

#### 2.4.1. Statistical Analysis

Data are presented as mean ± SD of six biological replicates (each with three technical replicates). Statistical significance was determined by Student’s *t*-test followed by Benjamini–Hochberg FDR correction for multiple comparisons between goji from NX and each of the other six cultivation areas (NM, GS, XJ, QH, XZ, and HB). FDR-adjusted *p*-values less than 0.05 were considered significant. RDA was conducted using Canoco 5.0 to examine and quantify the correlation between environmental factors and bioactive compounds as well as the antioxidant capacity of goji, with outcomes displayed on the x and y axes.

#### 2.4.2. UPLC-QTOF-MS Data Analysis

The raw LC-MS/MS data were converted to mzXML format using ProteoWizard software (version 3.0). Data processing, including peak alignment, retention time correction, and peak area extraction, was performed using MS-DIAL (version 4.60). The detailed processing parameters are provided in Supplementary Table S1. In the extracted-ion features, only variables with more than 50% of nonzero measurement values in at least one group were retained. The identification of metabolites was performed by comparing accuracy *m/z* value (<10 ppm) and MS/MS spectra with an in-house database established by available authentic standards (Wekemo Tech Group Co., Ltd., Shenzhen, China).

Multivariate statistical analyses, including principal component analysis (PCA) and orthogonal partial least squares discriminate analysis (OPLS-DA), were carried out using the Wekemo Cloud Platform (https://www.bioincloud.tech, accessed on 15 May 2025), which employs *R*-based algorithms for computation and visualization. Specifically, PCA and OPLS-DA were performed using the ropls package (version 1.18.8) within the R-based MetaboAnalystR framework (R version 3.1.3). To prevent overfitting and assess model validity, a permutation test (*n* = 1000) was performed during OPLS-DA modeling. Models with Q^2^ > 0.5 and permutation *p* < 0.05 were considered statistically significant and robust, indicating good predictive ability and no overfitting. Differential features in pairwise comparisons were screened though the integrated criteria of VIP values (>1) from OPLS-DA, absolute Log_2_Fold change (|Log_2_FC| > 0.585, chosen to prioritize biologically meaningful differences), and FDR-adjusted *p* < 0.05. These differential features were then subjected to KEGG pathway enrichment analysis using MBRole 2.0 (http://csbg.cnb.csic.es/mbrole2/, accessed on 15 May 2025), and pathway enrichment was assessed using a hypergeometric test with *p* < 0.05 considered significant. Features mapped to significantly enriched pathways were defined as discriminative markers. For these markers, retention time, accurate mass, and characteristic MS/MS fragments were manually verified to ensure identification reliability.

## 3. Results

### 3.1. Bioactive Constituents and Antioxidant Capacity of Goji from Different Cultivation Areas

**Figure 2:**
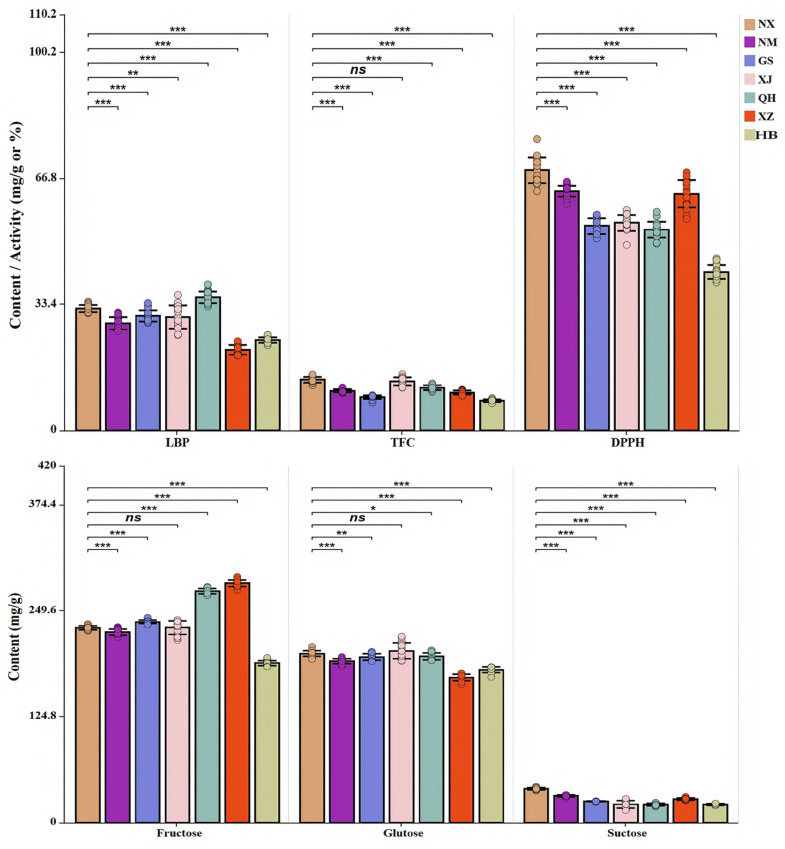
Comparative analysis of bioactive components and antioxidant activity in goji berries across seven cultivation areas. Each bar represents the mean ± SD (*n* = 18 per region, comprising six biological replicates each with three technical replicates). Statistical significance was determined by Student’s *t*-test comparing each region (NM, GS, XJ, QH, XZ, and HB) individually against NX. *p*-values were adjusted using the Benjamini–Hochberg FDR correction and are indicated by asterisks (* *p* < 0.05; ** *p* < 0.01; *** *p* < 0.001; ns, not significant; FDR-adjusted).

The key bioactive compounds, including LBP, TFC, and soluble sugars (fructose, glucose, sucrose), as well as antioxidant capacity via the DPPH assay were determined to evaluate the diversity of nutritional and functional profiles in goji from seven cultivation areas. As shown in Table S2, with data expressed as mean ± SD (*n* = 18 per region; six biological replicates, each measured in triplicate), both bioactive compounds and antioxidant activity exhibited significant geographical variations. The statistical analysis between NX and the other six regions are further shown in the differential abundance bar plot (Figure 2).

Goji from NX demonstrated the highest DPPH radical scavenging capacities and significantly exceeded that of all other regions (*p* < 0.01). NM and XZ followed, with no significant difference between them. QH goji exhibited the highest LBP content, significantly higher than that in second-ranked NX goji (*p* < 0.01). An apparent difference in TFC was also observed, with XJ and NX goji exhibiting higher content, and there was no significant difference (*p* > 0.05) between them. Phytochemical analyses further revealed regional characteristics in sugar compositions. Specifically, XZ and QH goji showed significantly increased fructose content than NX samples (both *p* < 0.01). In contrast, HB goji exhibited the lowest content of total soluble sugar (comprising fructose, glucose, and sucrose) among all regions.

### 3.2. The Relationship Between Environmental Factors, Bioactive Compounds, and Antioxidant Capacity of Goji from Different Cultivation Areas

RDA was employed to assess this association (Figure S1). The overall model explained 88.8% of the total variation (*R*^2^ = 0.888). Key factors that significantly influenced the chemical diversity of goji across regions included rainfall, daily maximum temperature, UV radiation, latitude, and days with large diurnal temperature variations (≥17 °C), which accounted for 26.7%, 21.1%, 19.9%, 13.9%, and 6.4% of the variation, respectively. Each of the principal axes mentioned was statistically significant (*p* < 0.01). Among these, rainfall, temperature, and UV radiation were identified as the top three influential factors.

We further analyzed the relationship between the bioactive compounds, antioxidant activity, and environmental factors across seven climatic regions of goji (Figure S1). LBP, TFC, fructose, glucose, sucrose, and antioxidant activity (DPPH) were negatively related to rainfall. Accordingly, regions with high rainfall, such as HB Julu and XZ Lhasa, exhibited lower levels of these compounds. Notably, in the extremely arid regions (GS and XJ), the level of these components remained moderate to low. In addition, the soluble sugar content (fructose and sucrose) and antioxidant activity (DPPH) showed a significantly negative correlation with the daily maximum temperature. Consistent with this, the elevated levels of soluble sugar were observed in QH and XZ, the regions with the lowest daily maximum temperature.

### 3.3. UPLC-QTOF-MS Metabolomic Analysis of Goji from Different Cultivation Areas

#### 3.3.1. Overview of the Metabolic Profiling in Goji

An untargeted metabolomic strategy based on UPLC-Q-TOF/MS was developed to comprehensively analyze the metabolites of goji from seven geographic origins. The total ion chromatograms (TICs) of representative goji samples are shown in Figure S2A,B. Both positive and negative ionization modes revealed a large number of ions in all samples, demonstrating the effectiveness of our analytical method for profiling the metabolome of goji berries. The reliability of the metabolomics platform was confirmed by multiple QC metrics. QC samples showed highly overlapping TICs in both ionization modes (ESI^+^ and ESI^−^; Figure S3A,B) and Pearson correlation analysis (r > 0.9 for all QC pairwise comparisons; Figure S3C,D). Furthermore, RSD analysis revealed that >80% of metabolic features detected in QC samples exhibited RSD ≤ 30% (Figure S3E,F).

**Figure 3:**
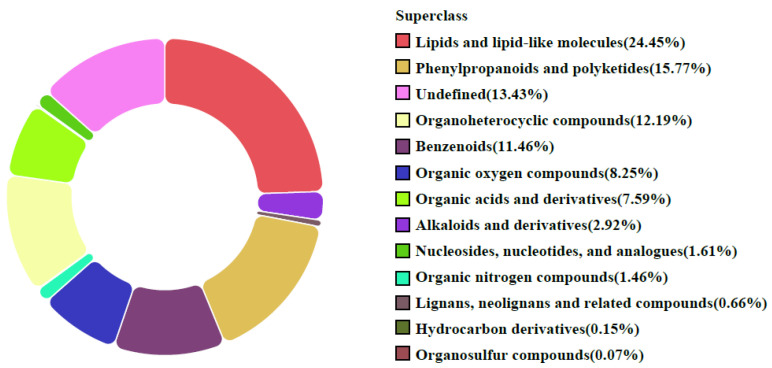
Classification of the 1364 putatively annotated mass spectral features in goji from seven producing regions.

After data processing and database matching against an in-house library and HMDB, 1364 mass spectral features were putatively annotated from combined positive and negative ion modes. Among these features, lipids and lipid-like molecules constituted the largest proportion (24.45%), followed by phenylpropanoids and polyketides (15.77%), organoheterocyclic compounds (12.19%), benzenoids (11.46%), organic oxygen compounds (8.25%), organic acids and derivatives (7.59%), alkaloids and derivatives (2.92%), nucleotides and derivatives (1.61%), and organic nitrogen compounds (1.46%). The remaining were undefined (13.43%) (Figure 3). This dataset provides a comprehensive foundation for further exploration of the goji metabolome.

It is worth noting that, despite some differences observed in the metabolite fingerprints of goji from seven production areas, the comparable TICs profiles was still a challenge for geographical discrimination. To resolve this complexity, we employed multivariate statistical analysis to reduce the data dimensionality and extract the most relevant differential features, integrating analysis of the positive and negative ionization results.

#### 3.3.2. Multivariate Analysis for Geographical Discrimination

**Figure 4:**
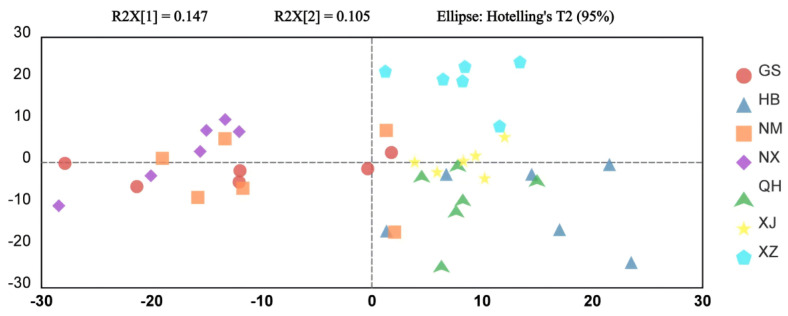
PCA score plot of goji berries from seven producing regions.

PCA was firstly executed and distinct clustering patterns were revealed across the seven production regions, which aligned with the regional climatic zones (Figure 4). Samples from semi-arid regions (NX, NM, GS) distributed predominantly in the second and third quadrants and showed substantial overlap. Moreover, they could not be distinguished from XJ samples along PC2. Although XZ and QH were both located at high-altitude environments, they showed clear separation along PC2 and were positioned in the first and fourth quadrants, respectively. HB goji were clearly differentiated from other samples along PC1. Overall, the PCA provided an initial overview of the metabolic variation, revealing some grouping trends among the samples. To more effectively identify the metabolic features associated with geographical origins, a supervised approach of OPLS-DA was subsequently employed.

**Figure 5:**
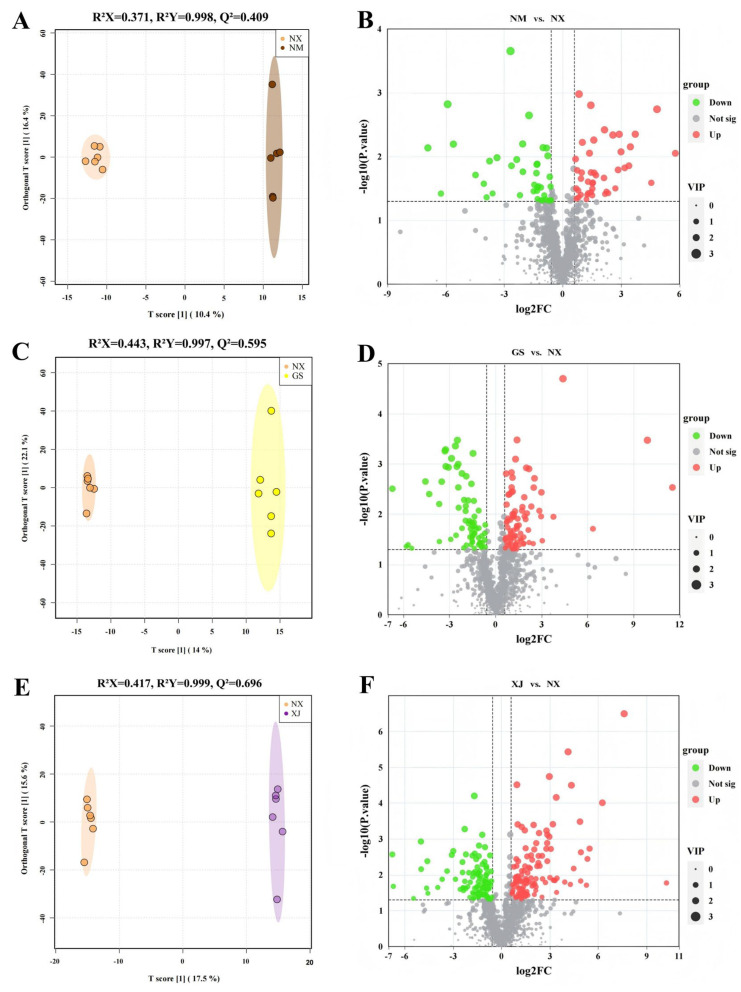

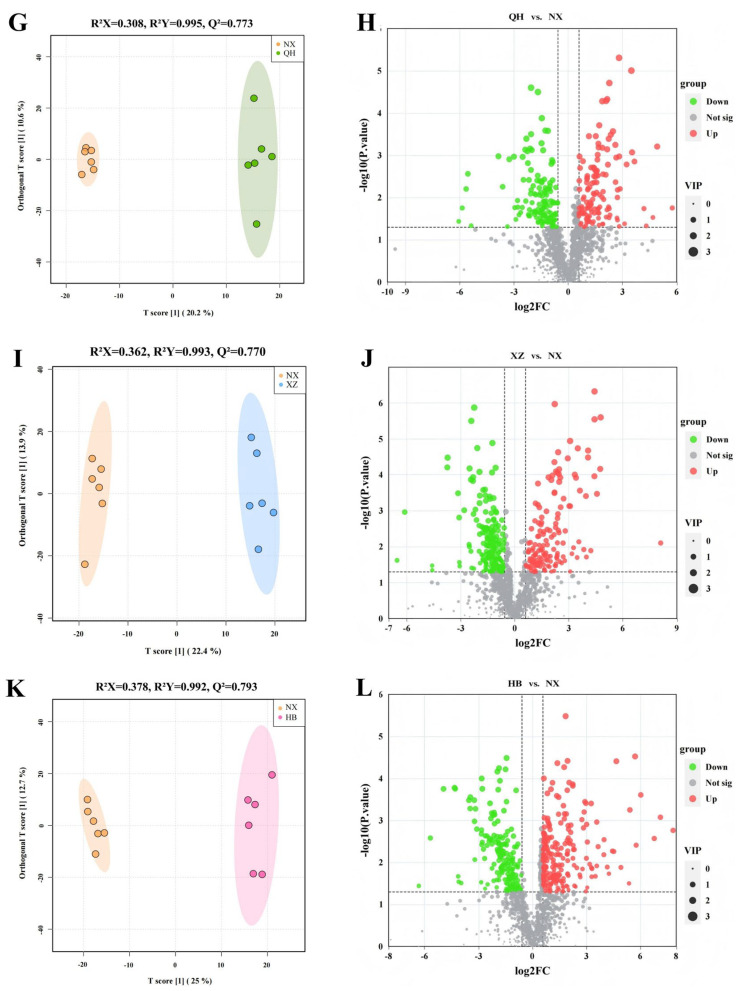
(**A**,**C**,**E**,**G**,**I**,**K**): OPLS-DA model plots for the comparison groups NX vs. non-NX (NM, GS, XJ, QH, XZ, and HB, respectively); (**B**,**D**,**F**,**H**,**J**,**L**): volcano plots that present the number of differentially metabolites in NX vs. non-NX (NM, GS, XJ, QH, XZ, and HB, respectively).

Given that Zhongning of Ningxia is the geo-authentic producing region of goji [2], we established this region as the reference in our OPLS-DA to systematically evaluate the metabolic discrimination against the other six major production regions (NM, GS, XJ, QH, XZ, and HB). The supervised approach effectively maximized inter-regional differences, as evidenced by clear separations in all pairwise comparisons (Figure 5A,C,E,G,I,K). The OPLS-DA models demonstrated strong explanatory power, with R^2^Y > 0.995 and predictive trends with Q^2^ > 0.4 [22]. Permutation testing (*n* = 1000) confirmed the validity of five out of six models, with Q^2^ > 0.5 and permutation *p* < 0.05 (Table S3). The NX vs. NM model showed lower predictive ability (Q^2^ = 0.409) and a non-significant permutation test (*p*= 0.238), suggesting that this model, though visually discriminative, did not meet the statistical criteria for reliability. Therefore, the NX vs. NM comparison was interpreted with caution and excluded from subsequent analyses as well as biological discussion. Based on these validated OPLS-DA models, differential features were considered using the following criteria: VIP scores > 1.0 to prioritize origin-related features; |log_2_FC| > 0.58 to identify substantive quantitative differences compared to the NX group (Figure 5D,F,H,J,L); and a univariate statistical significance of *p* < 0.05.

### 3.4. Identification and Functional Analysis of Key Discriminative Markers

Differential features identified from the five valid pairwise comparisons were subjected to KEGG pathway enrichment analysis. Features mapped to 10 significantly enriched pathways (*p* < 0.05) were considered as candidate ions and putatively annotated (Table S5). Through accurate mass (<10 ppm) and MS/MS spectra matching, the candidate ions were putatively identified as 49 key discriminative markers after removing duplicate (Table S6). Among them, 39 were detected in negative ion mode and 10 in positive ion mode. The mass spectrum of quercetin was elucidated in detail (Figure S4), with others shown in Figure S5.

#### 3.4.1. Metabolite Difference Between GS and NX

**Figure 6:**
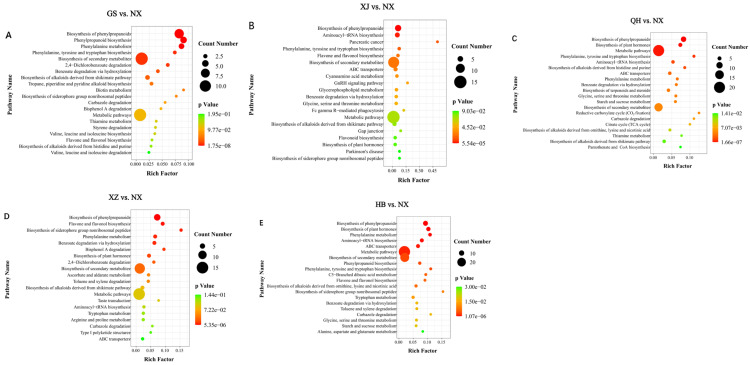
(**A**–**E**) KEGG enrichment of differential features between the comparison groups (GS/XJ/QH/XZ/HB vs. NX). Each bubble represents a metabolic pathway. The abscissa and bubble size together indicate the magnitude of the pathway’s impact factor, with a larger size denoting a larger impact. The color of the bubbles corresponds to the *p*-value from the enrichment analysis, where a redder color indicates a higher degree of enrichment.

Differential metabolites from the NX vs. GS comparison were significantly enriched in the biosynthesis of phenylpropanoid, phenylalanine metabolism, phenylalanine/tyrosine/tryptophan biosynthesis, and the biosynthesis of secondary metabolites pathway (Figure 6A and Table S4). Including nine, four, two, and 10 metabolites (Table S5), after excluding cross-pathway metabolites, six metabolites were upregulated in GS compared to NX, while 10 exhibited downregulation (Table S6).

NX goji exhibited enhanced phenylpropanoid metabolism, with eight out of nine metabolites significantly elevated (Table S5). Additionally, significantly elevated metabolites in the secondary metabolite biosynthesis pathways were observed in NX compared to GS, with tropic acid showing a 52-fold (log_2_FC = −5.69) increase (Table S5). Conversely, GS goji showed significantly elevated content of metabolites in the phenylalanine metabolism (e.g., *N*-acetyl-l-phenylalanine) and tryptophan biosynthesis (e.g., quinic acid).

#### 3.4.2. Metabolite Difference Between XJ and NX

Integration of *p*-value and rich factor, the differential metabolites between the NX and XJ regions were mainly concentrated in the biosynthesis of phenylpropanoids, aminoacyl-tRNA biosynthesis, pancreatic cancer pathway, and flavone and flavonol biosynthesis (Figure 6B and Table S4), including six, four, one, and two metabolites (Table S5). After removing cross-pathway duplicate metabolites, a total of six metabolites were upregulated in XJ compared to NX, while six exhibited downregulation (Table S6).

Similarly, NX goji accumulated two-thirds of phenylpropanoid pathway metabolites at significantly higher levels than XJ goji (Table S5). In contrast, XJ goji showed elevated levels of three key metabolites involved in aminoacyl-tRNA biosynthesis, namely serine, proline, and asparagine. Additionally, XJ goji exhibited significantly higher accumulation of quercetin, a key flavonoid in the flavone and flavonol biosynthesis pathway, with levels more than 1200-fold higher (log_2_FC = 10.23; Table S5).

#### 3.4.3. Metabolite Difference Between QH and NX

In this research, differential pathway enrichment (*p* < 0.05) between NX and QH groups revealed fundamental metabolic differences, prominently in phenylpropanoid and plant hormone biosynthesis, metabolic pathways, phenylalanine/tyrosine/tryptophan biosynthesis, and aminoacyl-tRNA biosynthesis (Figure 6C; Table S4), including eight, five, 20, three, and four metabolites, respectively (Table S5). After removing duplicates, 12 metabolites showed increased levels in QH versus NX, while 12 others decreased (Table S6).

Although NX goji accumulated higher levels of five out of eight phenylpropanoid intermediates, QH Plateau cultivars showed a marked 11-fold increase in daidzein biosynthesis (log_2_FC = 3.49; Table S5). In addition, NX goji exhibited increased levels of 50% of the metabolites in the general “Metabolic pathways”, with pantothenic acid and 5-hydroxyindole-3-acetic acid (5-HIAA) showing the highest fold changes. Notably, quinic acid accumulation was further enhanced in QH goji, with levels nearly 54-fold higher than those in NX within the tryptophan biosynthesis pathway (log_2_FC = 5.75; Table S5), exceeding the elevation observed in GS goji.

#### 3.4.4. Metabolite Difference Between XZ and NX

In the NX vs. XZ comparison, metabolites showing differential accumulation were primarily enriched in the biosynthesis pathways of phenylpropanoids, flavone, and flavonol, as well as siderophore group nonribosomal peptides (Figure 6D and Table S4), including seven, three, and two metabolites (Table S5). After removing duplicates, with a total of four metabolites increased in XZ compared to NX, while six metabolites decreased (Table S6).

In our study, NX goji exhibited a greater number of upregulated metabolites in the phenylpropanoids biosynthesis pathway, with five of seven metabolites, such as tryptophan, 4-hydroxybenzoic acid, and 2,3-dihydroxybenzoic acid, showing elevated levels than those from XZ (Table S5). In comparison, XZ goji displayed a different metabolic pattern along the phenylpropanoid pathway, which was reflected by the significantly accumulated levels of salicylic acid. Additionally, the content of quercetin was approximately 272-fold higher in XZ (log_2_FC = 8.10; Table S5) and daidzein levels was also upregulated compared to NX goji.

#### 3.4.5. Metabolite Difference Between HB and NX

Differentially abundant metabolites were significantly enriched in four major metabolic pathways, namely biosynthesis of phenylpropanoids, biosynthesis of plant hormone, aminoacyl-tRNA biosynthesis, and phenylalanine metabolism (Figure 6E; Table S4). These pathways contained nine, seven, six, and five differential metabolites (Table S5), which corresponded to 12 metabolites with increased abundance and eight with decreased in HB goji after deduplication (Table S6).

In the present study, we found that NX goji consistently showed a greater number of upregulated metabolites along the phenylpropanoid pathway. Among which, two-thirds of the metabolites, including 2,3-dihydroxybenzoic acid, tryptophan, and sinapic acid, were present at significantly higher levels compared to HB goji. In contrast, four out of seven metabolites in the plant hormone biosynthesis pathway accumulated at higher levels in HB goji, such as salicylic acid and anthranilic acid (Table S5). Meanwhile, five out of six aminoacyl-tRNA synthetase substrates (e.g., glutamic acid, serine, leucine; Table S5) exhibited higher levels in HB goji. In addition, all metabolites in the phenylalanine metabolism pathway were upregulated in HB goji.

## 4. Discussion

### 4.1. Regional Variation in Bioactive Constituents and Antioxidant Capacity

The levels of bioactive constituents (including TFC, LBP, fructose, glucose, and sucrose) and the antioxidant capacity of goji berries varied significantly across the seven cultivation regions. This reginal diversity offers valuable insights for the targeted applications of goji in the food and nutraceutical fields. For instance, NX goji exhibited the highest DPPH radical scavenging capacities, consistent with prior antioxidant assessments using multi-mechanisms (e.g., ABTS, FRAP) [23]. Its extract has been successfully applied in meat storage to improve sensory properties and oxidative stability [5]. The higher TFC in goji from the regions NX and XJ provides an important chemical basis for flavonoid-targeted products development. Given that LBP is well-established as a primary functional compound with extensive health benefits [4,5], this regional identity supports the use of LBP as a critical indicator for assessing the bioactive potential and overall quality of goji. In particular, the high LBP content in QH goji makes it a premium ingredient for wellness products aimed at improving immunity and skin health. Fructose, the predominant sugar in goji, was more abundant in XZ and QH, enhancing their appeal for food and nutritional applications due to its contribution to desirable sweetness [24]. In contrast, HB goji are low in sugar, which aligns well with the growing market for low-glycemic products.

### 4.2. Environmental Drivers of Bioactive Accumulation and Antioxidant Capacity

RDA further elucidated that climatic factors, particularly rainfall, temperature, and UV radiation, were the key drivers of the regional metabolic variation. Specifically, rainfall was negatively correlated with the levels of key bioactive compounds and antioxidant capacity. One possible explanation is that excessive rainfall, especially when followed by prolonged humid conditions, may promote pathogen infestations, which could in turn affect nutrient accumulation and fruit quality. However, it should be noted that other correlated climatic factors (e.g., HB Julu had the highest rainfall and the lowest UV radiation) likely also play a role. Conversely, extreme drought conditions can also impair plant growth [25], where inadequate soil moisture appears to constrain nutrient synthesis, resulting in generally moderate to low contents of these components. Although the impact is not uniform across all components, water scarcity remains an adverse factor for fruit growth in these regions.

The elevated soluble sugar levels in QH and XZ goji are likely associated with cold tolerance under low temperatures, consistent with the negative correlation with daily maximum temperature. However, given that QH and XZ also have the highest UV levels and elevations, UV radiation is known to induce the accumulation of soluble sugars as a protective mechanism, while high-altitude stress can trigger osmotic adjustment [26]. Therefore, the metabolic profiles of QH and XZ goji likely reflect a combination of these co-varying factors rather than a single driver. The elevated DPPH scavenging capacity in XZ goji can be attributed to the combined effects of intense UV radiation, and high-altitude and low-latitude conditions, which induce greater oxidative stress. This enhanced antioxidant capacity likely represents a survival strategy against cumulative oxidative damage from multiple environmental challenges [27]. Larger diurnal temperature variations have been reported to promote daytime photosynthesis and nutrient accumulation, while cooler nighttime temperatures reduce respiratory losses [28]. Accordingly, our findings further confirmed that these conditions enhanced the storage of sugars and polyphenols, providing insights into how local environmental factors shape the nutritional and functional traits of goji from different regions.

### 4.3. Metabolic Profiling and Geographical Discrimination

Beyond these targeted bioactive components, untargeted metabolomics provided a broader view of regional metabolic variation. The PCA clustering pattern revealed that goji from NX, GS, NM, and XJ shared similar metabolites profiles, which were consistent with the observed similarities in certain morphological features (all have a wrinkled surface, as shown in the macroscopic image in Figure 1). This phenomenon may be attributed to the comparable ecological conditions and standardized cultivation practices. The clear metabolic separation between XZ and QH implied that metabolite diversity was shaped by multiple environmental factors beyond altitude, including the diurnal temperature range, precipitation patterns, and UV radiation. The pronounced clustering differentiation of HB goji is likely due to its low-altitude and semi-humid monsoon conditions, which are characterized by intense summer rainfall and fundamentally different growing environment. Notably, the OPLS-DA model did not reveal a robust metabolic distinction between NX and NM goji. This metabolic similarity tentatively suggests that the *daodi* zone may extend into neighboring Inner Mongolia, potentially due to shared ecological conditions. However, given the statistical limitations, this interpretation remains speculative and requires further validation.

### 4.4. Functional Implications of Discriminative Markers

A previous targeted metabolomics study on goji from three growing regions [10] identified region-specific metabolites but did not further explore their functional roles in stress adaptation. Expanding on this, the discriminative markers putatively identified in the present study (49 markers across seven regions) showed accumulation patterns that varied significantly across regions, reflecting distinct environmental pressures and holding potential for functional food and nutraceutical applications.

In contrast to the well-irrigated cultivation systems typical of NX, GS goji are cultivated in a more arid environment with lower rainfall and higher light intensity. The activation of phenylalanine metabolism and tryptophan biosynthesis likely represents an adaptive mechanism to cope with drought and oxidative stress. Research has shown that *N*-Acetyl-l-phenylalanine enhances plant drought tolerance by regulating phenylalanine metabolism, thereby promoting flavonoid synthesis and mycorrhizal colonization, and ultimately maintaining biomass under drought conditions [29]. Quinic acid exhibits a synergistic effect with *N*-Acetyl-l-phenylalanine on enhancing plant resistance against oxidative damage and osmotic stress induced by drought and UV radiation, maintaining cellular homeostasis [30]. These findings may assist in the selection of goji varieties suited to arid zones.

In XJ goji, the enrichment of serine, proline, and asparagine involved in aminoacyl-tRNA biosynthesis reflects an alternative stress response under arid conditions. These amino acids have been reported to play roles in plant adaptation to arid conditions, with proline acting as a compatible osmolyte, while serine and asparagine are involved in nitrogen metabolism and stress signaling [31]. In addition, quercetin, a flavonol and potent antioxidant, is significantly elevated in XJ goji. It may contribute to reactive oxygen species scavenging and osmotic adjustment, especially under arid conditions [32]. Beyond its role in plants, its antioxidant and antimicrobial properties have attracted interest in food science, where it is considered a potential ingredient for functional foods and natural preservatives [33].

The harsh high-altitude environment of QH promotes secondary metabolite synthesis in goji, developing a dual survival strategy against mountain stressors. The upregulation of isoflavonoids (e.g., daidzein) may contribute to antioxidant defense by scavenging UV-induced reactive oxygen species, protecting plant cells from oxidative damage under high-altitude UV stress [34], while key intermediates (e.g., quinic acid) support drought resistance through antioxidant defense.

XZ goji, also from a high-altitude region, exhibited a distinct metabolic profile. The significantly accumulated levels of salicylic acid align with the higher rainfall in this region, suggesting a potential adaptive role in plant stress tolerance and fungal resistance under humid conditions [35]. This natural defense capacity may offer potential for low-pesticide management strategies under humid conditions. Quercetin, which was markedly higher in XZ goji, is well established for its functions in UV protection, cold tolerance, and antioxidant defense under high-altitude conditions [32,36]. Daidzein was also upregulated, reflecting typical plateau adaptation responses. Overall, the distinctive metabolic profile of XZ goji suggests its promise for highland nutraceutical development and stress-resilient breeding.

In HB goji, the high-rainfall, humid environment promoted the accumulation of salicylic acid and anthranilic acid. These two metabolites, which are involved in the plant hormone biosynthesis pathway, may coordinate resource allocation under high-rainfall conditions by regulating signal transduction and auxin polar transport, effectively balancing plant growth and defense [35,37]. Meanwhile, the upregulation of multiple aminoacyl-tRNA synthetase substrates likely modulates protein synthesis and energy metabolism via the TCA cycle and nitrogen assimilation [31], alleviating nutrient stress and compensating for reduced photosynthesis caused by shaded and humid conditions. In addition, phenylalanine metabolism is known to play a key role in responses to various environmental stresses, including but not limited to drought [29]. Its enrichment in the high-rainfall region HB suggests that this pathway may play additional roles in adapting to local stresses, including pathogen infection, oxidative stress, and other challenges associated with high summer temperatures, excessive moisture, and relatively insufficient sunlight. However, further validation is required to confirm this conclusion.

Across the five pairwise comparisons, NX goji consistently exhibited a greater number of upregulated metabolites along the phenylpropanoid pathway. These phenylpropanoids play critical roles in plant defense against combined biotic and abiotic stress, acting as essential components of cell walls, phytoalexins against pathogens, and protectants against UV radiation. Their presence in fruits may contribute to maintaining fruit integrity and bioactivity during development and post-harvest storage [38,39,40]. Among these uniformly elevated metabolites, 4-hydroxybenzaldehyde demonstrates tyrosinase inhibitory effects through mechanisms involving Schiff base formation or copper chelation, making it a potential agent for preventing enzymatic browning in fruits and vegetables [41]. 2,3-Dihydroxybenzoic acid shows strong α-amylase inhibition (IC_50_ = 0.74 mg/mL), supporting its suitability for postprandial blood sugar control [42]. Tryptophan, which was higher in NX goji than in most other regions, plays a dual role. In plants, it alleviates oxidative damage and maintains ion homeostasis under salt stress [43]. From a nutritional perspective, it serves as a precursor of gut microbial metabolites that enhance intestinal barrier integrity and promote uric acid excretion [44]. Therefore, the elevated tryptophan levels in NX goji may not only support stress tolerance but also enhance its potential as a functional food for gut health and hyperuricemia management. In specific comparisons, NX goji also exhibited higher levels of certain compounds. For instance, compared to GS goji, NX goji showed higher levels of tropic acid, a precursor of defensive alkaloids involved in plant stress responses [45,46]. Compared to QH goji, NX goji exhibited increased levels of pantothenic acid and 5-hydroxyindole-3-acetic acid (5-HIAA), which play roles in primary metabolism and stress resilience [47,48].

These region-specific metabolic traits not only elucidate stress adaptation mechanisms but also serve as diagnostic markers for assessing local environmental pressures. Moreover, the key metabolites within these pathways represent promising intervention targets for enhancing crop resilience through targeted breeding or metabolic engineering.

In addition to their roles in stress adaptation, these markers exhibited distinct accumulation patterns across regions, suggesting their potential as chemical fingerprints for geographical authentication of goji berries. For instance, quinic acid may serve as an indicator for goji berries from water-stressed environments such as GS and QH, while salicylic acid could be associated with high-rainfall regions like XZ and HB. HB goji are further characterized by the higher accumulation of amino acids (e.g., leucine and proline). In contrast, NX goji are distinguished by the consistent upregulation of key phenylpropanoid markers (e.g., 4-hydroxybenzaldehyde, 2,3-dihydroxybenzoic acid, tryptophan) across multiple comparisons. Together, these findings support the broader objectives of improving crop resilience, formulating precision agriculture strategies, and contributing to the authenticity and quality assessment of goji products.

## 5. Conclusions

Consequently, this study revealed significant regional variations in the bioactive compounds, antioxidant capacity, and metabolic profiles of goji berries from seven core cultivation areas. The quantitative analysis of nutritional and functional traits, including TFC, LBP, soluble sugars, and DPPH, which highlighted region-specific quality characteristics shaped by local climatic factors, also provided a basis for the differentiated development of goji berries. Untargeted metabolomics identified 49 discriminative markers. They reflect the active metabolic adaptation of goji to diverse environments and serve as chemical fingerprints for geographical authentication. It should be noted that the RDA in this study focused on climatic and geographical factors but did not include soil properties (e.g., pH, mineral composition), which have been reported to influence secondary metabolite accumulation in goji berries. Future studies incorporating soil physicochemical parameters are needed to provide a more comprehensive understanding of environmental drivers shaping goji metabolic profiles. Additionally, studies with larger sample sizes should be conducted to validate the stability and generalizability of chemical markers for origin identification, particularly for discriminating between goji berries from NM and NX. Moreover, deeper investigations into the molecular mechanism of plant stress resistance should be conducted.

## Figures and Tables

**Table 1:** Environmental factors of the seven goji cultivation areas in China.

Index	Zhongning (NX)	Linhe (NM)	Yumen (GS)	Jinghe (XJ)	Golmud (QH)	Lhasa (XZ)	Julu (HB)
DAT [°C]	20.31	18.82	17.70	20.90	13.87	14.84	23.08
Days with ∆T [≥17 °C, d]	65	75	118	30	65	3	9
DMAT [°C]	37.39	36.11	35.61	41.28	34.78	27.50	41.61
DMIT [°C]	−1.72	−2.39	−8.28	−2.78	−9.89	−0.39	3.11
Accumulated temperature [≥10 °C, °C]	2037.39	1755.78	1592.13	2155.44	956.11	980.55	2571.51
Rainfall [mm]	154.44	94.48	24.89	46.22	40.14	361.19	728.97
Sunlight hours [h]	3066	3300	3246	2700	3350	3005	2235
UV [MJ/m^2^]	490.28	500.50	552.74	487.94	580.68	578.42	433.39
RH [%]	36.41	28.05	21.86	31.65	21.67	47.95	50.56
Alt. [m]	1193	1041	1527	330	2809	3650	65
Lat. [°N]	37.49	40.75	39.83	44.65	36.42	29.65	37.22
Long. [°E]	105.69	107.42	97.57	82.88	94.90	91.12	115.03

DAT: daily average temperature; days with ∆T: days with large diurnal temperature variations; DMAT: daily maximum temperature; DMIT: daily minimum temperature; UV: ultraviolet radiation; RH: relative humidity; Alt.: altitude; Lat.: latitude; Long.: longitude.

## Data Availability

The original contributions presented in the study are included in the article/Supplementary Materials; further inquiries can be directed to the corresponding author.

## Supplementary Materials

The following supporting information can be downloaded at: https://www.mdpi.com/article/10.3390/metabo16050326/s1. Figure S1. RDA plot showing the relationship between environmental factors, bioactive compounds, and antioxidant capacity of goji from seven regions; Figure S2. Overlaid TICs of representative goji samples from seven goji cultivation areas; Figure S3. Overlaid TICs, Pearson correlation analysis, and RSD analysis of QC samples; Figure S4. MS/MS analysis of quercetin; Figure S5. MS/MS spectra of the 49 putatively identified discriminative metabolites; Table S1. MS-DIAL parameters for data processing; Table S2. Bioactive constituents and antioxidant properties of goji berry samples; Table S3. Validation of OPLS-DA models for comparing NX with other goji berry cultivation regions; Table S4. KEGG enrichment analysis of differential features between NX and other cultivation areas; Table S5. Putatively annotated metabolites mapped to significantly enriched pathways; Table S6. Putatively identified discriminative markers in goji berry samples.
